# IFEM model framework for the accreditation of training sites for emergency medicine specialists

**DOI:** 10.1186/s12245-025-01001-3

**Published:** 2025-10-08

**Authors:** Andrew Singer, Simon Chu, Anantharaman Venkataraman, Nicholas Jouriles, Arif Alper Cevik, James Kwan

**Affiliations:** 1 Australian Government Department of Health and Aged Care, GPO Box 9848 MDP L4N, Canberra, ACT 2601 Australia; 2 Australian National University Medical School, Acton, ACT Australia; 3 University of Adelaide, Lyell McEwin Hospital, Elizabeth Vale, Playford, SA Australia; 4 Department of Emergency Medicine, Singapore General Hospital, Singapore, Singapore; 5 Department of Emergency Medicine, Northeast Ohio Medical University, Rootstown, OH USA; 6 Departments of Internal Medicine, Emergency Medicine Section, College of Medicine and Health Sciences, United Arab Emirates University, Al Ain, United Arab Emirates; 7 Department of Emergency Medicine, Tawam Hospital, Al Ain, United Arab Emirates; 8 Department of Emergency Medicine, Tan Tock Seng Hospital, Singapore, Singapore; 9 Yong Loo Lin School of Medicine, National University of Singapore, Singapore, Singapore

**Keywords:** Emergency medicine, Accreditation framework, Graduate medical education, Clinical training, Accreditation standards

## Abstract

The International Federation for Emergency Medicine (IFEM) has developed model curricula for undergraduate education, graduate education and continuing professional development in Emergency Medicine. Graduate education is delivered in various settings, including training sites based in a clinical Emergency Department. In order to ensure the quality of the training that is delivered at these sites, mature training systems have a system that accredits these sites for training purposes. This paper presents a model framework for this accreditation process that may be used by national societies to develop their own process. This paper also includes a comparison of this framework with the accreditation standards of established programs in IFEM member societies.

**Supplementary Information:**

The online version contains supplementary material available at 10.1186/s12245-025-01001-3.

## Introduction

### Development of graduate medical education curriculum

The International Federation for Emergency Medicine (IFEM), through the Core Curriculum and Education Committee, has developed model curricula for Undergraduate (basic) Medical Education in Emergency Medicine (EM) [1], Graduate (postgraduate or specialist) Medical Education (GME) in Emergency Medicine [2] and Continuing Professional Development post training [3]. For GME, this provides the structure to assist in the development of a comprehensive training program for specialists in EM. In most countries, this is developed by a society or organization of specialists in EM. In line with these model curricula, the accreditation framework here provides a model for National EM societies to develop their own system of accreditation of training sites used as part of their GME program.

### Purpose of accreditation

In the context of this document, accreditation refers to an official body giving authority or sanction to an individual, a training site or an organization when recognized standards have been met with respect to training for emergency medicine specialists.

In medical care, there are many areas where standards may be set by an official body. This can include standards of practice of an individual, standards with regards to the design and equipping of a health facility, standards of care provided or standards relating to the performance of a facility in whole or in part. Accreditation provides a process whereby the performance of an entity can be assessed against these standards and then appropriately recognized.

For a specialty such as EM, recognized by the appropriate official body in that jurisdiction, a GME curriculum in EM provides the pathway and standards with which to train and certify specialist practitioners. Training is provided through a combination of formalized teaching across a range of modalities and supervised clinical experience in an appropriate environment, such as a hospital emergency department. The supervision and teaching may be provided by members of the specialist organization or by other practitioners with suitable training and experience. Accreditation provides a quality assurance mechanism by which a specialist organization or other authority can assess and ensure optimal delivery of the teaching and training of the curriculum.

Standards and the accreditation based on these can be applied on a number of levels. The General Medical Council of the United Kingdom (GMC) described this through their Education Strategy and Quality Improvement Framework [4]. It is based on the principles of proportionality, accountability, consistency, transparency and targeting. In the GMC Quality Improvement Framework, the governance of the quality of training is described at three levels:

- Quality Assurance – this refers to “the policies, standards, systems and processes in place to maintain and enhance the quality of medical education and training.” This would normally be carried out by a national authority (such as a Department of Health or a national medical council) tasked with recognising specialties, medical schools, their curricula and training programs.
- Quality Management – this refers to the process used to accredit and monitor the performance of training sites (named “Local Education Providers” in the GMC Quality Improvement Framework). It should be based on appropriate standards set by the relevant body. Namely, that would be the organization responsible for defining the standards of the specialty in a jurisdiction (for example, a specialist EM organization).
- Quality Control – this refers to the role training sites have in ensuring they are delivering training that meets the standards set through the quality management process. This would be a responsibility of the senior education leaders, supervisors and trainers working at these training sites.

### Aim of framework

The aim of this model framework is to describe an approach to accreditation at the “Quality Management” level described above. It specifically covers the accreditation of training sites, and should be able to be integrated with the other levels of quality improvement as required.

The World Federation for Medical Education (WFME) has produced a series of documents, “Global Standards for Quality Improvement” in medical education, referring to Basic Medical Education [5], Postgraduate Medical Education [6] and Continuing Professional Development (CPD) of Medical Doctors [7]. For those organizations seeking to undertake a “Quality Assurance” level process in the absence of established national standards and processes, these standards provide a useful starting point to assess such a training program.

The framework described here is primarily aimed at the “Quality Management” level as described above. It is derived from work done for the Accreditation of Specialist Medical Training Sites Project, commissioned by the Australian Health Ministers’ Advisory Committee (AHMAC) through its Health Workforce Principal Committee [8]. It consists of a hierarchy of Domains, Standards and Criteria that are applicable to training sites associated with any specialist training program. In describing the framework, examples that may apply to a specific EM specialist training program are included.

As well as describing the standards that need to be met for effective training, the framework also needs to include the authority under which the standards are applied, and the system that governs, assesses and monitors the accreditation process and its outcomes (for example, see Table 1 in Taber, et al.) [9].

**Table 1:** Accreditation domains, standards, criteria and EM examples for EM training sites (adapted from AHMAC) [8]

Domain	Standard	Criterion	EM Examples
1 Promotes the health, welfare and interests of trainees	1.1 Governance, safety and quality assurance of training sites.	1.1.1 A training site has clear governance structures which support: (a) optimal safety and quality; (b) education and training; (c) workplace health, safety and welfare of trainees; and (d) trainee participation in governance.	*Role of ED Director*,* training site Director of EM Training.* *Hospital and ED management structures.* *Trainee representation in governance of the training site program*,* such as on committees.* *Quality assurance of the training site program.*
		1.1.2 Trainee management structures are effective.	*Number of trainees at the site; links between training*,* employment and service provision; rosters.* *Policies relating to trainee welfare such as bullying and harassment.* *Where relevant*,* selection and/or employment of trainees.* *Networked training with other facilities.*
		1.1.3 There are appropriate quality assurance processes in place for clinical care.	*QA/QI committees*,* clinical audit*,* review of patient pathology and radiology results.*
	1.2 Infrastructure, facilities and educational resources	1.2.1 There are appropriate educational resources and these are available to trainees.	*Access to textbooks*,* journals*,* library and/or IT services and eLearning*,* including decision support software.*
		1.2.2 The training site provides a physical environment that supports trainees.	*Trainee study/rest space*,* meeting/tutorial rooms*,* workspaces/IT access for clinical applications.*
2 Ensures trainees have appropriate knowledge, skills and supervision to deliver quality patient care	2.1 Department specialist staffing and supervision	2.1.1 There are appropriate staff (EM specialists and/or others, as appropriate) to ensure effective supervision of trainees at all times	*Ratio of supervisors to trainees*,* supervisor qualifications and training in both EM and teaching methods*,* roster coverage*,* on-call arrangements.*
		2.1.2 Supervisory staff understand their roles and responsibilities and are supported in their supervisory roles	*Supervisor training and support*,* including time “off the floor” for training support.* *Clear communication of goals*,* objectives and expectations for training.*
		2.1.3 The designated Director of Training (or equivalent) is supported in the role and is available to trainees	*Work time available to teach*,* manage and support trainees*,* space available for private conversations.*
	2.2 The provision of clinical experience and work is relevant	2.2.1 The training site provides the appropriate breadth and volume of clinical experience	*ED acuity and casemix*,* attendances*,* admission rates; distinct patient groups e.g. trauma*,* paediatrics*, etc. *Trainee exposure to and responsibility for patients as measured by the above parameters.*
3 Supports a wide range of educational and training opportunities aligned to the curriculum requirements	3.1 Education, training, teaching and learning opportunities	3.1.1 Teaching and learning opportunities in the workplace are targeted and enable exposure to the breadth of experience in the working environment	*Patients that trainees will treat*,* graduated and escalating levels of independence and responsibility*,* procedures*,* supervision of junior staff*,* non-ED or external rotations*,* all mapped to the curriculum.*
		3.1.2 Structured education programs and continuing medical education sessions are accessible to trainees	*Didactic teaching*,* tutorials*,* regional and national EM education programs*,* journal clubs*, etc. *Alignment of program with GME curriculum.* *A system for “in-house” trainee assessment against the curriculum*,* including workplace-based assessment.* *Support in trainee preparation for external assessments.*
	3.2 Multidisciplinary clinical support services and equipment	3.2.1 Information on relevant supporting services and specialties to support the delivery of the specialty service	*Nursing and allied health ED staff*,* on-site specialty services available*,* off-site referral networks*,* opportunities for interprofessional learning.*
		3.2.2 Equipment is available to provide the specialty service	*Examples include ED sonography*,* critical care therapies and monitoring*,* airway adjuncts and aids*,* slit lamps and other specialized equipment used in ED.*
	3.3 Research opportunities are promoted and facilitated	3.3.1 The training site facilitates and supports specialty specific research activity	*Departmental EM research*,* academic tracks available*,* mandated research as per the curriculum and/or training site program.*
	3.4 Accreditation by others, supporting information	3.4.1 The training site is accredited by other recognised accreditation bodies	*Accreditation of hospital for clinical services*,* quality and safety; accreditation of training site programs in other specialties.*

### Model framework

The model framework consists of a series of related elements:

1. Domains, standards and criteria for accreditation.
2. Governance and delivery of accreditation process.
3. Accreditation teams – membership and training.
4. Outcomes of accreditation.

### Accreditation domains, standards, criteria

Table 1 lists model generic standards for training sites. Examples of how they might apply specifically to an EM training program are included. They also broadly align with the WFME Quality Improvement Standards for Postgraduate Medical Education [6], which are designed to apply to a national training program.

Areas such as selection and admission into training, formal teaching, or allocation of rotations may be the responsibility of the training site and/or the national training program. There should be relevant standards that apply to these areas for the national training program, or additional standards may need to be developed where these responsibilities rest with the training site.

As shown in the “EM Examples” column in Table 1, further guidance can be provided through examples or detail around the evidence required to demonstrate that the standards are being met.

### Governance and delivery of accreditation

The governance and delivery of the training site accreditation includes the elements of accreditation standard development, development and supervision of accreditation processes, decisions on process outcomes and delivery of the accreditation process itself.

#### Development of accreditation standards

Whilst the model framework here provides suggested domains, standards and criteria for the accreditation of training sites, a national training program in a jurisdiction is expected to modify and potentially add to these such that they correctly align with the training structures at the national and local level, local EM models of care and any external clinical or regulatory requirements. Whilst there may be external frameworks and processes that can be used for local purposes, they should still be adapted to suit local needs and requirements. As these directly relate to the delivery of the curriculum, this would normally be done through the body responsible for overseeing the curriculum, such as a national EM specialty organization.

Other expected features include a clear statement of the intended scope and purpose of the accreditation process, general information for the facilities being accredited, definitions for any specific terminology that relates to the specialist program’s processes. Terminology that is understood and agreed in the wider context should be used wherever possible.

#### Development and supervision of accreditation process and decisions on outcomes

A committee or similar structure responsible for supervising the delivery of accreditation should be established. This committee may be within the body responsible for the national training program, or part of an external body specifically tasked with accreditation of training for one or more specialties. It should ideally include expertise on the curriculum, the delivery of accreditation, training sites, supervisors, trainees, consumers/laypeople, other members of the healthcare team (such as other medical specialties, nurses or allied health) and regulators (where relevant). Where accreditation has a direct impact on trainee employment, employer representation may be relevant as well. Members need to be aware of and able to appropriately manage perceived or actual conflicts of interest, such as decisions involving a training site a member has a direct interest in. There should be clear terms of reference for the committee that address the above issues.

Other issues such a committee may need to manage are:

- Sources of information – the relative role and nature of site visits, questionnaires, formal and informal reporting.
- Accreditation cycle – in most instances, accreditation determinations will occur on a regular cycle (e.g., once every five years). Within that cycle, there should some form of progress reporting (e.g., annually) to keep the committee up to date on changes and developments at the site. The cycle may need to be varied where issues are identified at a site that need closer monitoring or changes matched to a specific timeframe. Site visits may be part of the start or end of a cycle, or part of closer monitoring where necessary.
- Decisions on outcomes, with communication of this to the site and other relevant parties, such as a government health authority.
- Membership and training of accreditation teams (where site visits are part of the program).
- Quality improvement of the accreditation process.
- Matching the standards and processes to community expectations and national needs, such as required workforce skills and geographical distribution of trainees.

### Accreditation processes

Potential models for accreditation processes include:

1. Through and by the committee managing accreditation for the national EM body (e.g. Australasian College for Emergency Medicine).
2. Through an external body with expertise in accreditation of training, potentially combined with accreditation in other health areas through a streamlined process (examples include the UK General Medical Council and the USA Accreditation Council for Graduate Medical Education).
3. Self-assessment by sites, with review by an oversight committee.

These models are not exclusive to each other, and how they are used will be determined by a combination of available resources (including human resources), complexity and size of existing training structures and external regulatory requirements.

Where the process uses a formal visit to the training site, it should involve an assessment of the site against the accreditation standards, include observation of the clinical training environment and any training facilities, provide opportunities to interview key people in the local training process (such as the Director of Training, supervisors, support staff) and receive confidential feedback from trainees at the site.

The goals of the visit and the standards that will be applied should be transparent and publicly available. This should also include a description of the metrics used to determine that a standard is met.

### Accreditation teams – skills and training

Regardless of how the accreditation process is undertaken, where the process involves a visit by a team of assessors external to the training site, the following skills should be represented in the team (more than one skill may be held by any individual team member):

- Experience in the accreditation process and assessing the standards.
- Understanding and experience in the training curriculum and its local delivery (e.g., Directors of Training from other sites, Supervisors).
- Specialist knowledge in EM practice.
- Understanding of the trainee experience (e.g., other trainees or recent graduates from a program).
- An ability to have an objective and independent view of the conduct of the process (e.g., a jurisdictional or other external member).
- An opportunity for team development (e.g., junior team members as observers).

All team members should be briefed on the accreditation standards and their appropriate application, understand the decision-making process, its implications and their responsibilities and obligations with regard to the process. They should also understand and be able to deal with conflicts of interest if and when they arise.

### Outcomes of accreditation

Under most existing accreditation systems, a successful outcome would result in some form of certification allowing the site to conduct the training program for a specified period of time (if all standards are satisfied, at least the length of the cycle between visits, where visits are used). There may also be consideration of the training capacity of the site (how many trainees can be supported at a particular site). Where standards are only partially satisfied, there should be a process that allows for delivery of training to continue with remediation of any issues, such as reducing the length of the cycle with a further review through a site visit or by a local education supervisor, or by having specific conditions that must be satisfied to allow ongoing certification.

In the event the program fails to meet the accreditation standards and remediation has been unsuccessful or not possible, a pre-defined method should exist to close the program and transfer any faculty and trainees.

There should also be an ability to deal with disputes over the determined outcome, such as documented appeals or review processes.

### Thematic analysis of GME accreditation standards in emergency medicine

To support member organizations in creating their own accreditation standards and structures based on the model, we conducted a thematic analysis of the accreditation standards used for GME in Emergency Medicine across Australia, New Zealand, Canada, the United Kingdom, and the United States of America. This is detailed in Appendix 1.

This demonstrates where the standards in the IFEM Model Framework have similarities to existing national accreditation frameworks for EM training sites, as well as demonstrating where these standards fit within an international accreditation framework from the World Federation for Medical Education. As well as multiple similarities across domains, the analysis also demonstrates where there are differences in scope and delegated responsibilities. As a model framework, the IFEM Model Framework is deliberately set at a higher level than the comparison frameworks and this is evident in the analysis. This is detailed in the Commentary section of Appendix 1.

## Conclusion

This model framework provides the basis on which IFEM member societies can create a mechanism to assess and monitor the quality of training provided in their programs, as well as regulate training sites for the benefit of all our patients.

## Supplementary Information

Supplementary Material 1.

## Data Availability

No datasets were generated or analysed during the current study.
