# Supplementary material for: IFEM model framework for the accreditation of training sites for emergency medicine specialists
Source: Int J Emerg Med. 2025 Oct 8;18:196. doi: 10.1186/s12245-025-01001-3 (PMC12505791; doi:10.1186/s12245-025-01001-3)
Supplement: Supplementary file 1 — Supplementary Material 1. [file 12245_2025_1001_MOESM1_ESM.docx]

**Thematic Analysis of Training Site Accreditation Standards in Selected IFEM Member Organizations**

To assist member organizations in developing their own accreditation standards and structures based on the model, the tables below are a thematic analysis of the accreditation standards used for GME in Emergency Medicine in Australia and New Zealand, Canada, United Kingdom and United States of America. They are compared using the domains in the World Federation for Medical Education Global Standards for Quality Improvement for Postgraduate Medical Education.

Whilst these domains are designed to assess a national GME program, they are relevant in their application to the accreditation of training sites as well. The source documents for comparison are:

1. WFME: World Federation for Medical Education - Postgraduate Medical Education: WFME Global Standards for Quality Improvement – The 2015 Revision, 2015
2. Australasian College for Emergency Medicine (ACEM, Australia and New Zealand): AC549 – FACEM Training Program Site Accreditation – Requirements v1.05, January 2020
3. Royal College of Physicians and Surgeons of Canada (RCPSC, Canada): Standards of Accreditation for Residency Programs in Emergency Medicine (122A), July 2020
4. American Council for Graduate Medical Education (ACGME, USA): ACGME Program Requirements for Graduate Medical Education in Emergency Medicine, July 2021
5. General Medical Council (GMC, UK): Promoting Excellence: standards for medical education and training, January 2016

The WFME standards consist of Areas (Whole numbers), Sub-areas (decimal points) and Standards (see separate summary below for the original standards).

*Summary of Postgraduate Medical Education WFME Global Standards 2015*

1. Mission and Outcomes
   1. Mission
      stated, publicly available, based on community need, outcomes-based, safe for trainees, innovative.
   2. Professionalism and Professional Autonomy
      collaborate with government, academic freedom.
   3. Educational Outcomes
      defined, knowledge, skills, attitudes, life-long learning, community need, professional behaviour, publicly available.
   4. Participation in Formulation of Mission and Outcomes
      collaboration with appropriate stakeholders.
2. Educational Program
   1. Framework of Program
      outcome-based, systematic, transparent, practice-based, integrated methods, equality/equity, trainee-centred, supervision and feedback, ethical considerations.
   2. Scientific Method
      program founded on medical research and epidemiology, trainee outcomes, EBM.
   3. Program Content
      theoretical content, skills, clinical practice, communication, ethics, medico legal, management, patient safety, practitioner resilience.
   4. Program Structure, Composition and Duration
      including compulsory and optional components, integrate theory and practice, national regulations, exposure to community/health system interaction, competency-based achievement.
   5. Organisation of Education
      who has responsibility and authority for educational settings and process, stakeholder representation, broad range of trainee experiences, multi-site.
   6. The Relation between PME and Service
      note apprenticeship model, integrate and compliment training and service.
3. Assessment of Trainees
   1. Assessment Methods
      formative versus summative, transparent, matched to framework and content, reliable, valid, fair, appealable.
   2. The Relation between Assessment and Learning
      assessment compatible with outcomes, promotes trainee learning, includes feedback.
4. Trainees
   1. Admission Policy and Selection
      related to mission, balances educational capacity, policy-based, transparent and equitable, appealable, stakeholder involvement, regular review.
   2. Number of Trainees
      proportionate to training opportunities, supervisor capacity, resources available, adapted according to need.
   3. Trainee Counselling and Support
      academic counselling, monitoring trainee progress, trainee support, confidentiality, career guidance, trainee involvement.
   4. Trainee Representation
      design of mission, outcomes, program, working conditions, evaluation, program management.
   5. Working Conditions
      balance between training and service, appropriate remuneration, equal participation, transparency.
5. Trainers
   1. Recruitment and Selection Policy
      policy-based, expertise, balance of responsibilities, clear duties, matched to mission and program, relevant qualifications, recognition of participation, use of educational expertise.
   2. Trainer Obligations and Development
      time available, faculty development, periodic evaluation, trainer support, trainee-supervisor ratio.
6. Educational Resources
   1. Physical Facilities
      trainee study space, access to literature, ICT access, equipment for practical training, safe learning environment, regularly updated.
   2. Learning Settings
      sufficient to support learning, relevant number and case-mix of patients, hospital and community-based.
   3. Information Technology
      internet access, effective use, allows self-directed learning, communication, access to patient data.
   4. Clinical Teams
      teamwork, interprofessional learning.
   5. Medical Research and Scholarship
      research opportunities, balance between training and research, access to facilities.
   6. Educational Expertise
      appropriate use in planning, implementation and evaluation of program.
   7. Learning in Alternative Settings
      trainee access, including international.
7. Program Evaluation
   1. Mechanism for Program Monitoring and Evaluation
      routine monitoring, clear mechanism, address all aspects, feedback loop, stakeholder involvement, transparency.
   2. Trainer and Trainee Feedback
      seek feedback from trainers, trainees, employers.
   3. Performance of Qualified Doctors
      monitor performance of completed trainees, feedback loop.
   4. Involvement of Stakeholders
      involvement in monitoring and evaluation and providing feedback.
8. Governance and Administration
   1. Governance
      program meets regulations, documented completion of program, quality improvement.
   2. Academic Leadership
      appropriate leadership and staff, evaluation of performance.
   3. Educational Budget and Resource Allocation
      budget responsibility, adequate resource allocation.
   4. Administration and Management
      appropriate administrative staff, quality assurance and review.
   5. Requirements and Regulations
      program meets national regulations.
9. Continuous Renewal
   regular review, update of all aspects, fix identified problems, appropriate resources for process.

**How to read the Table**

For each set of compared standards, each cell refers to the section or paragraph numbering used in the source document, where it covers the same theme (standard).

Where a reference refers to a higher-level standard, it assumes that all sub-standards are included.

A standard in a source document may relate to multiple themes in the WFME standards, as reflected in multiple entries in the analysis.

Source document standards and requirements are matched to sub-areas under the WFME standards.

**Commentary**

In general, most of the WFME standards are matched by similar standards in the compared frameworks. This includes common references under the domains of the educational program, assessment and support of trainees, supervisors of trainees (“Trainers” in the WFME framework) and educational resources.

Differences relate to the following issues:

1. Differences in scope: In some examples (UK and Canada in particular), these are standards that apply to all GME programs, not just EM (though with some EM-specific additions) whereas in others they are specific to EM training (Australia and New Zealand, USA). Other differences in scope relate to whether the focus of the standards is exclusively on training sites (IFEM, UK, Australia and New Zealand) or where an institution has responsibility in areas such as the Mission and Outcomes of the training program or its Framework (USA). Some of these are described within a national curriculum (Australia and New Zealand, UK), or institution-wide standards (Canada) rather than specific to training site accreditation.
2. Delegated responsibilities: There are differences between programs as to the role that the training site plays in selection of trainees into the training program, and how this may link to employment at the facility level. For some programs, this is all under the responsibility of the training site. Other programs may operate through trainee selection at a national or regional level. For the IFEM Model Framework, these are optional activities and so are not included but should be covered if the training site has this responsibility.
3. Detailed Frameworks: Compared to the IFEM Model Framework, the tabled programs have multiple references that can be categorised within any one area or sub-area of the WFME Standards. Some of this reflects differences in emphasis and organisation of each set of standards. The IFEM Model Framework is aimed to be a high-level set of standards. In developing a local accreditation framework, more specific detail may be included. It is important to ensure that the standards continue to focus on what is important for quality of training, and on what is feasible and based on evidence (where available).

**Tables 1-8: Thematic Analysis of Selected GME Accreditation Standards for Training Sites compared to WFME Standards (see above for explanation)**

| 1. Mission and Outcomes  *Note: This is expected to be described at a higher level, applying to a national training program which then guides the accreditation of training sites.* | | | | | |
| --- | --- | --- | --- | --- | --- |
| WFME | USA (ACGME) | Canada (RCPSC) | Australia/New Zealand (ACEM) | UK (GMC) | IFEM Model Framework |
| 1.1. Mission | Int.A., Int.B. | Addressed in Institution standards @ PMGE level | 1.1.1.1, |  | This domain is expected to be described at a higher level, applying to a national training program which then guides the accreditation of training sites. |
| 1.2. Professionalism and Professional Autonomy | Int.A., Int.B., IV.B.1.a), VI.B. | 3.2.1.3, 3.2.2, 3.2.4.2 |  |  |  |
| 1.3. Educational Outcomes | Int.A., Int.B., IV.B., VI.B. | 3.1, 3.2.1, 3.2.2.5, 3.2.3.1, 3.2.4.4 | Covered in ACEM Curriculum which is a separate document | R3.15, S5.2, |  |
| 1.4. Participation in Formulation of Mission and Outcomes |  | 1.2.1, 1.2.2, 1.2.3 | 1.1.1.6, | R2.3, |  |

| 2. Educational Program | | | | | |
| --- | --- | --- | --- | --- | --- |
| WFME | USA (ACGME) | Canada (RCPSC) | Australia/New Zealand (ACEM) | UK (GMC) | IFEM Model Framework |
| 2.1. Framework of Program | IV.A., IV.B., IV.C.3. | 3.1, 3.2.1, 3.2.2, 3.2.4.1, | 2.2.1.1, 3.1.2.1, | R1.9, S2.3, R2.17, S5.2, R5.1, R5.9a, R5.9b, | Covered through a national program run by the relevant society |
| 2.2. Scientific Method | IV.A.6., IV.B.1.c).(2) | 3.1, 3.2.3, | 3.1.2.1, |  |  |
| 2.3. Program Content | IV.A.5., IV.B., IV.C.2., IV.C.4., IV.D.3., VI.A.1.(b) | 3.2.1, 3.2.2, 3.2.3, 3.2.6, | 2.2.1.1, 3.1.2.1, 3.1.2.2, | R1.14, | 3.1.2 |
| 2.4. Program Structure, Composition and Duration | Int.C., Int.C1., I.B.4.c), IV.A.2., IV.A.4., IV.C.1., IV.C.3, IV.C.4. | 3.2.1, 3.2.2, 3.2.4.2, | 2.2.1.1, 3.1.2.1, 3.1.2.2, 3.1.2.3, | R1.13, R2.12, R2.15, R5.9a, | 3.1.2 |
| 2.5. Organisation of Education | Int.C.1., I.A., I.B.3, I.B.4.c), II.A., II.A.4. | 1.2.1, 2.1.1, 2.3.1, 3.4.3, | 2.1.3.4, 1.1.2.1 | S2.3, R2.6, R4.5, | 3.1.2 |
| 2.6. The Relation between PME and Service | IV.B.1.a)-b), IV.B.1.d), VI.F.1., VI.F.3. | 3.2.2.5, 3.2.4, 6.1.2 | 1.1.1.5, 3.1.1.1, 1.1.1.3, 1.1.3.1 | R1.9, R1.12, R1.16, R3.12, R5.9h, | 3.1.1 |

| 3. Assessment of Trainees | | | | | |
| --- | --- | --- | --- | --- | --- |
| WFME | USA (ACGME) | Canada (RCPSC) | Australia/New Zealand (ACEM) | UK (GMC) | IFEM Model Framework |
| 3.1. Assessment Methods | V.A., VI.A.2. | 3.4.1, 3.4.2, 3.4.4 | 1.2.1.2, 3.1.1.2, | R5.10, R5.11 | 3.1.2 |
| 3.2. The Relation between Assessment and Learning | V.A.1., V.A.1.d), V.A.3. | 3.4.2 (particularly 3.4.2.5), 3.4.3, 3.4.4, | 3.1.1.2, | R1.18, | 3.1.2 |

| 4. Trainees | | | | | |
| --- | --- | --- | --- | --- | --- |
| WFME | USA (ACGME) | Canada (RCPSC) | Australia/New Zealand (ACEM) | UK (GMC) | IFEM Model Framework |
| 4.1. Admission Policy and Selection | II.A.4.a).(9), III.A., III.C. | 6.1.1.1, | ACEM  Regulation G |  | Optional |
| 4.2. Number of Trainees | III.B | 3.2.4.4, | 2.1.1.3, | R2.20, | Optional |
| 4.3. Trainee Counselling and Support | I.D.1.h), II.A.4.a).(13)-(15), VI.B.4., VI.C. | 1.2.1.3, 3.4.2, 3.4.4, 5.1.2, 5.1.3.2-5.1.3.5, 6.1.2 | 1.1.1.2, 1.1.1.4, 1.1.2.2, 1.1.2.3, 2.1.3.5, | R1.1, R1.10, R1.15, R1.21, R2.12, R2.14, R2.15, R2.16, S3.1, R3.1, R3.2, R3.3, R3.5, R3.7, R3.11, R3.13, R3.14, R5.12, | 1.1.2 |
| 4.4. Trainee Representation | II.A.4.a).(10), VI.C.1.e) | 1.2.1.2, 6.1.1.2, 7.1.1.3, | 1.1.1.6, 1.1.2.3, 1.1.3.2 | R1.22, R2.3, | 1.1.1 |
| 4.5. Working Conditions | I.B.4.c).(1), I.D.2., II.A.4.a).(7), IV.A.4.a), VI.B.4., VI.C., VI.D., VI.E.1., VI.F. | 3.2.4.3, 3.2.4.5, 5.1.2.2, 5.1.2.3, | 1.1.1.2, 1.1.1.5, 1.1.2.3, | R1.12, R1.16, R3.10, R3.12, | 1.1.2, 3.1.1 |

| 5. Trainers | | | | | |
| --- | --- | --- | --- | --- | --- |
| WFME | USA (ACGME) | Canada (RCPSC) | Australia/New Zealand (ACEM) | UK (GMC) | IFEM Model Framework |
| 5.1. Recruitment and Selection Policy | II.A.4.a).(4)-(6), II.B.1., II.B.3. | 4.2.1, 7.1.2 | ACEM  Regulation G | R1.7, R2.20, S4.1, R4.1, | 2.1.1 |
| 5.2. Trainer Obligations and Development | II.A.2.a), II.B.2., II.B.4., IV.D.2.  VI.A.2. | 3.3.1, 5.1.1, 7.1.1, 7.1.2, | 2.1.1, 2.1.2, 2.1.3.3, | R1.7, R1.8, R1.22, R2.3, R2.11, R2.14, S4.2, R4.2, R4.4, | 2.1.2, 2.1.3 |

| 6. Educational Resources | | | | | |
| --- | --- | --- | --- | --- | --- |
| WFME | USA (ACGME) | Canada (RCPSC) | Australia/New Zealand (ACEM) | UK (GMC) | IFEM Model Framework |
| 6.1. Physical Facilities | I.D.1.b), I.D.1.d), I.D.2.b)-e), I.D.3. | 4.1.3, 4.1.3.2, 4.1.3.3, 4.1.3.4, 4.1.3.7, 4.1.1.11, 4.1.2.12 | 1.1.1.2, 1.1.1.3, 1.1.3.1, 1.2.2.1, 3.2.1.1, 3.2.2.1 | R1.19, | 1.2.2, 3.2.2 |
| 6.2. Learning Settings | I.B.4., I.D.1., I.D.1.f), I.D.1.g), I.D.4., VI.F. | 2.3.1, 4.1.1, 4.1.2.4, 4.1.2.5, 4.1.2.7, 4.1.2.3, 5.1.1.1, 5.1.2, 5.1.3.1, | 1.1.1.1, 1.1.1.3, 1.1.3.1, 1.2.1.1, 2.2.1.2, 2.2.1.3, 3.1.1.1, 3.1.2.3, 3.1.2.4, 3.2.1.1, 3.2.1.2, 3.2.1.4, 3.2.2.2 | S1.1, S1.2, R1.1, R1.4, R1.12, R1.13, R1.19, R2.11, R2.14, R3.6, R5.9c, | 3.1.1, 3.1.2 |
| 6.3. Information Technology | I.D.1.b).(7), I.D.3., IV.B.1.d).(1).(g)-(k) | 4.1.3.3, 4.1.3.6, | 1.2.1.1, 1.2.1.2, 1.2.1.3, 1.2.2.1, 3.2.1.2, 3.2.1.3, | R1.20, R5.9d, | 1.2.1, 1.2.2 |
| 6.4. Clinical Teams | I.D.1.c), I.D.1.e), I.E., VI.E.2, VI.E.3. | 4.1.2.2, 4.1.2.3, 4.1.2.4, 4.1.2.9, 4.1.2.10, 4.1.2.13, 4.1.5.2 | 2.1.1.1, 2.1.1.2 | R1.17, R5.9e-f, | 2.1.1, 3.2.1 |
| 6.5. Medical Research and Scholarship | IV.D. | 3.2.5, 4.2.1.4, 7.1.2.4, | 3.3.1, | R3.8, | 3.3.1 |
| 6.6. Educational Expertise | IV.C.3., VI.F. | 1.1.3.6, 4.1.5, 7.1.1.4-7.1.1.6, | 3.1.2.4, | R4.5, | 2.1.1, 2.1.2 |
| 6.7. Learning in Alternative Settings | IV.B.1.f).(1).(d), IV.C.5.b) | 4.1.2.1, 4.1.2.8, | 2.2.1.3, 3.1.1.3, 3.2.1.4, |  | 3.1.1 |

| 7. Program Evaluation | | | | | |
| --- | --- | --- | --- | --- | --- |
| WFME | USA (ACGME) | Canada (RCPSC) | Australia/New Zealand (ACEM) | UK (GMC) | IFEM Model Framework |
| 7.1. Mechanism for Program Monitoring and Evaluation | I.B.3., V.C. | 1.1.2.3, 7.1.1.1, 7.1.1.2, 9.1.1, 9.1.2.5, 1.2.2.4, | 1.1.1.1, AC550 Accreditation Process Guide | R2.4, R2.7, | 1.1.3 |
| 7.2. Trainer and Trainee Feedback | V.B. | 3.3.1.5, 7.1.1.3, 9.1.2.3, 9.1.2.7, | 1.1.1.6, 1.1.2.3, | R1.1, R1.5, R1.22, R2.1, | 1.1.2, 1.2.2 |
| 7.3. Performance of Qualified Doctors | V.C.3. | 9.1.1.4, | Regulation B | R2.5, | 3.1.2 |
| 7.4. Involvement of Stakeholders | VI.C.1.e).(1) | 1.2.3, 2.2.1, 9.1.2.1-9.1.2.2, | 1.1.3.2, | R1.22, R2.3, R2.8, | 1.1.1 |

| 8. Governance and Administration | | | | | |
| --- | --- | --- | --- | --- | --- |
| WFME | USA (ACGME) | Canada (RCPSC) | Australia/New Zealand (ACEM) | UK (GMC) | IFEM Model Framework |
| 8.1. Governance | I.A.1., I.B.1., I.B.2., II.A., V.A.3. | 1.1.1, 1.1.3, 1.2.1, 1.2.1.4, 1.2.2, 2.3.1, 9.1.2, | 1.1.1, 1.1.3.1, | R1.2, R1.6, S2.1, S2.2, S2.3, R2.1, R2.2, | 1.1.1 |
| 8.2. Academic Leadership | I.B.3.a), II.A., V.B.1. | 1.1.3, 2.3.2, | 1.1.2.1, 2.1.2.1, 2.1.2.3, 2.1.3.1, 3.3.1.4, | R2.2, | 1.1.1 |
| 8.3. Educational Budget and Resource Allocation | I.B.2., I.D. II.A. | 2.3.3, 4.1.4.1, | 2.1.2.2, 2.1.2.4, 2.1.3.2, 2.1.3.3, 2.1.3.5, | S2.3, R2.10, R4.3, |  |
| 8.4. Administration and Management | II.A.4., II.C., II.D. | 1.1.2, 1.2.2, 2.3.3, 4.1.3.5, 4.1.5, 8.1.1, | 1.1.2.1, 1.1.2.3, 1.1.3.1, 1.1.3.2, 2.1.3.6 |  | 1.1.2 |
| 8.5. Requirements and Regulations | II.A.4.a).(8), II.A.4.a).(11)-(13) | 3.1.1.1, 3.2.1.2, 3.2.2.4, 3.4.1.5, 3.4.3.3. | 2.1.3.4, 3.4.1, | S1.2, R1.11, R2.9, R2.19, R3.4, R4.6, | 1.1.2 |
| 9. Continuous Renewal | V.C. | 9.1.1, 9.1.2, 9.1.3, | 1.1.1.6, | R1.3, S2.1, R2.4, | 1.1.3 |
